# The G-NORM Scale: Development and Validation of a Theory-Based Gender Norms Scale

**DOI:** 10.1007/s11199-022-01319-9

**Published:** 2022-09-03

**Authors:** Erica Sedlander, Jeffrey B. Bingenheimer, Michael W. Long, Minati Swain, Rajiv N. Rimal

**Affiliations:** 1 Institute for Health and Aging, Department of Social and Behavioral Sciences, University of California, San Francisco, California, San Francisco, United States; 2 Milken Institute School of Public Health, Department of Prevention and Community Health, The George Washington University, Washington D.C., United States; 3 DCOR Consulting, Bhubaneswar, India; 4 Department of Health, Behavior and Society, Johns Hopkins Bloomberg School of Public Health, Baltimore, United States

**Keywords:** gender norms, social norms, scale development, measurement

## Abstract

Gender norms are increasingly recognized as important modifiers of health. Despite growing awareness of how gender norms affect health behavior, current gender norms scales are often missing two important theoretical components: differentiating between descriptive and injunctive norms and adding a referent group. We used a mixed-methods approach to develop and validate a novel gender norms scale that includes both theoretical components. Based on qualitative data, the theory of normative social behavior, and the theory of gender and power, we generated a pool of 28 items. We included the items in a baseline questionnaire among 3,110 women in Odisha, India as part of a cluster randomized controlled trial. We then ran exploratory factor analysis which resulted in 18 items. Using a second wave of data with the same sample, we evaluated psychometric properties using confirmatory factor analysis and structural equation modeling. The analysis resulted in two subscales with nine items each, “descriptive gender norms” and “injunctive gender norms.” Both subscales represent high internal validity with Cronbach’s alpha values of 0.81 and 0.84 and the combined scale has an alpha of 0.87. The G-NORM, gender norms scale, improves on existing measures by providing distinct descriptive and injunctive norms subscales and moving beyond individual attitudes by assessing women’s perceptions of community-level gender norms.

Gender norms are social norms defining acceptable and appropriate actions for women and men in a given group or society. They are embedded in formal and informal institutions, nested in the mind, and produced and reproduced through social interaction. They play a role in shaping women and men’s (often unequal) access to resources and freedoms, thus affecting their voice, power, and sense of self. (Cislaghi and Heisee, 2020, p. 9).

Inequitable gender norms are increasingly recognized as a direct threat to population health and a barrier to longer-term economic and social development (Connell, 2012; Doyal, 2000). Inequitable gender norms underlie deep-rooted unhealthy behaviors like female genital mutilation, intimate partner violence, and child marriage. In lower and middle-income countries, inequitable gender norms negatively affect uptake of a broad range of preventive behaviors including contraception use, perinatal services, cervical cancer screening, HIV testing, iron folic acid supplement use, and immunizations (Caal et al., 2013; Cianelli et al., 2008; Garrett & Barrington, 2013; Paudel et al., 2018; Sedlander et al., 2021; Singh et al., 2013).

However, this rapid increase in attention to addressing gender norms as a public health priority, has not been matched by a theoretical understanding of the multi-level construction of social norms as modifiable causes of health behavior. Many scales designed to assess gender norms have not adequately incorporated key theoretical constructs of social norms theory that have been shown to influence health behaviors (Moreau et al., 2019). The purpose of the present study is to develop and validate a gender norms scale that includes these social norms concepts.

## Social Norms Theory

*Descriptive norms* and *injunctive* norms are critical concepts in social norms theory (Cialdini et al., 1991). Perceived descriptive norms refer to individuals’ beliefs about what other people do (e.g., most girls in this community get married before the age of 18). Perceived injunctive norms, in contrast, are individuals’ beliefs about others’ approval or disapproval (e.g., most people in this community believe that girls *should* get married before they turn 18). Rimal & Real (2005) built upon prior social norms theories, the focus theory of normative conduct, and Fishbein and Ajzen’s (1975) theory of reasoned action, to develop the theory of normative social behavior (TNSB). In this revised theory, authors argue that injunctive norms strengthen or attenuate the relationship between descriptive norms and the outcome (an attitude or behavior). Therefore, measuring both separately provides important and unique information about how norms affect behavior (Lapinski & Rimal, 2005; Rimal & Lapinski, 2015). Additionally, the measurement of social norms, of which gender norms is a subcategory, requires explicit consideration of the referent group which could be family, friends, community, etc. (e.g., “most families I know believe that”) in which the norms operate (Rimal & Lapinski, 2015). Furthermore, when developing a gender norms scale, it is necessary to consider both social norms theory and gender theory.

## Theory of Gender and Power

According to Connell’s (1987) theory of gender and power, three constructs characterize the gendered relationship between men and women: (1) the sexual division of labor; (2) the sexual division of power which refers to control, authority, and coercion within heterosexual relationships; and (3) the structure of cathexis which depicts the often unequal roles and norms that dictate relationships between men and women. Connell describes local gender norms as “gender regimes” and “gender orders” at different levels of analysis (e.g., the school, the family, the state, and the street). Connell states that these higher order areas have normative expectations around how specific genders should behave, consistent with the definition of injunctive norms. Connell differentiates these norms from individual expectations and argues that many aspects of gender need to go beyond the individual and should be understood as collective, social practices (p. 139). More broadly speaking, scholars argue that gender is constructed and reinforced across levels of the socio-ecological model, including the macro-level of institutions as well as the micro-level of the household (Ridgeway & Correll, 2004; Risman, 2004).

## Gender Norms Measurement

Although several scales exist to measure gender norms, including the Gender Equitable Men (GEM) scale, the Participation in Household Decision-Making scale, the Support for Traditional Gender Roles scale, the Gender Norms instrument, and the Gender Norms Attitude subscale (EMERGE, 2021; Nanda 2011; Pulerwtiz & Barker, 2008; The C Change Program, 2018), none of these scales adequately incorporates the key theoretical constructs of social norms theory shown to influence health behaviors (Moreau et al., 2019). Specifically, they are missing two critical components: First, they do not differentiate between descriptive and injunctive norms. Second, they do not include a reference group (e.g., perceptions of friends, family, workplace, community, etc.) Instead, most existing instruments measure gender role *attitudes* and *beliefs* rather than social norms, perceptions of what others are doing or are expected to do.

One prominent example is the Gender Equitable Men (GEM) scale, a 35-item measure of men’s attitudes toward gender norms in Brazil which is separated into 2 domains: Inequitable Gender Norms (e.g., “I would be outraged if my wife asked me to use a condom”) and Equitable Gender Norms (e.g., “In my opinion, a woman can suggest using condoms just like a man can”). The GEM scale has also been validated in Uganda with one domain and 18 items (e.g., “It is ok for a man to hit his wife if she will not have sex with him (EMERGE, 2021; Pulerwitz & Barker, 2008).” Another example is the Gender Norms Attitude scale, which measures egalitarian beliefs about male and female gender norms with 14 items (two subscales: rights and privileges for men and equity for girls). An example from this scale is, “I would like my daughter to be able to work outside the home so she can support herself if necessary” (EMERGE, 2021; Nanda, 2011).

Though these measures have been critical to the field and bring us closer to measuring the construct of gender norms, they only measure individual attitudes and beliefs, missing out on what the target population believes are the norms within their community (what “others” believe and do). The measurement of social norms, of which gender norms is a subcategory, requires explicit consideration of the referent group (e.g., “most families I know believe that”) in which the norms operate (Rimal & Lapinski, 2015). However, current gender norms scales do not draw this distinction, and they do not differentiate between descriptive and injunctive norms. Two exceptions that do differentiate between descriptive and injunctive norms (although not in separate subscales) are the Global Early Adolescent Scale (GEAS), which measures the descriptive and injunctive gender norms around intimate relationships among young adolescent (ages 10–14; Moreau et al., 2019), and the Gender and Adolescence: Global Evidence (GAGE) program, which measures descriptive and injunctive gender norms among young adolescents (ages 10–12; Baird et al., 2019).

Another limitation of existing measures is their tendency to focus too narrowly on one domain rather than broader gender norms. One prominent example is the Sexual Relationship Power Scale (Pulerwitz et al., 2000), which focuses on the negotiation of sexual interactions between partners (e.g., “If I asked my partner to use a condom, he would get violent” and “My partner might be having sex with someone else.”). While the behavior is important and relevant to gender, it is rather narrow and does not measure social norms which requires a referent group (e.g., perceptions about what *other people* are doing or expect you to do).

## Current Study

In this paper, we describe the development of a gender norms scale that builds on previous gender norms scales in three specific ways: we identify a community-level referent group (e.g., “most families you know believe that”), distinguish between descriptive and injunctive norms, and include a broader set of items that represent gender norms that we identified from our prior qualitative research in Odisha, India (Sedlander et al., 2018; Sedlander et al., 2020). We report on the development and psychometric properties of the scale among women of reproductive age who completed the scale as part of an iron-folic acid promotion cluster-randomized controlled trial in Odisha, India (Yilma et al., 2020).

## Method

This study was approved by the George Washington University Institutional Review Board (IRB), Sigma Science and Research, an independent IRB located in New Delhi, India, and the Indian Council for Medical Research’s (ICMR’s) Health Ministry’s Screening Committee (HMSC).

### Study Setting

Odisha is a coastal state in eastern India where most households are situated in rural areas. Most heads of household are Hindu (95%) and 23% of households belong to a specific tribal culture. Almost a quarter of people in Odisha belong to the Scheduled Tribe (Mohindra & Labonté, 2010). Of the castes and tribes within Odisha, people who belong to the Scheduled Tribe have been the most marginalized followed by Scheduled Caste. While discrimination based on the caste system is illegal in India, people who belong to lower castes, like the Scheduled Tribe, continue to face worse health outcomes than their counterparts who belong to higher castes (Mohindra & Labonté, 2010). Women’s literacy rates illustrate existing caste/tribe disparities. In the whole state of Odisha, the female literacy rate is 64% but within the Scheduled Tribe population, literacy is only 41% and the Scheduled Tribe population is only 58% (International Institute for Population Sciences, 2017, NFHS, 2015–2016). To bridge disparities, the government of India promotes the social, economic, and educational interests of both Scheduled Castes and Scheduled Tribe (Article 46 of the Constitution of India, Government of India, 2012). The state is divided into 30 districts, one of which, Angul, is our focal district. In Angul, 70% of women are literate compared to 87% of men and 22% of women marry before age 18 (International Institute for Population Sciences, 2017, NFHS 2015–2016).

### Stages of Scale Development and Validation

We developed the gender norms scale in three stages: (1) formulation of items for the scale based on three sources: the collection and analysis of qualitative data in the study communities, review of the literature and expert input, and cognitive interviewing with draft scale items; (2) determining the dimensionality of the scale and identifying and removing poorly performing items by applying *exploratory factor analysis* to baseline questionnaire data; and (3) validation of the scale by applying *confirmatory factor analysis* to end line questionnaire data and examining associations with select sociodemographic and behavioral variables. All qualitative and quantitative data were collected as part of a larger study, the RANI project, which developed and conducted an impact evaluation of a social-norm based intervention to promote iron folic acid supplement use and reduce anemia among women of reproductive age in Odisha, India (Yilma et al., 2020).

#### Stage 1: Formulation of Scale Items

**Qualitative Data Analysis.** To ensure that the items we developed were relevant to the population where we tested them, we collected qualitative data in the project study communities. Between March and May 2018, we collected data from two blocks (administrative units below the district), Kishorenagar and Athamalik, in four villages. We completed a household listing of each geographic vicinity and participants were randomly sampled. Participants were visited at home by a female research assistant and asked to participant in a study about women’s lives and nutrition. We conducted 16 focus groups and 21 individual interviews (*n* = 148) stratified by age and gender with women of reproductive age, husbands, mothers-in-law in the community, and key informants. Interview guides covered general questions about what women do on a typical day, their concerns and aspirations, and roles in the family and community. To explore women’s social norms in a less personal way within the focus groups, we used vignettes, short stories about hypothetical characters that live in a rural village in Angul, India (Gourlay et al., 2014). Vignettes can also help examine if social sanctions exist and test emerging hypotheses about existing social norms (Institute for Reproductive Health, 2017). Four researchers, two from India and two from the United States, with backgrounds in nutrition, gender, and maternal and child health analyzed transcripts using NVivo v.12 to identify gender norms and then met with a larger group of researchers to discuss emerging themes. For a full description of the qualitative methods including participant demographics, see Sedlander et al., (2020).

The three domains reflected in this scale were the most salient gender norm themes: (1) gendered expectations, (2) household power and decision-making, and (3) “other oriented.” Each sub domain taps into a different part of the larger construct of gender norms. Gender expectations refers to the values, behaviors, and beliefs that a society considers appropriate for men and women. Power and control refer to the imbalance between disadvantaged groups’ access to resources and power over others (Connell, 1987). Household decision making refers to who makes decisions about important aspects of the home, such as large household purchases and visits from family and friends. “Other oriented” refers to the socialization of women to put others first at the expense of their own health, needs, and desires (Upadhyay et al., 2014).

We used Connell’s theory of gender and power throughout the course of this study both inductively and deductively. For example, we kept these domains front and center as we were creating the original items and the three domains that emerged from the qualitative data also map onto the three constructs within Connell’s theory of gender and power. “Gendered expectations” aligns with “the sexual division of labor” or the allocation of types of work based on an individual’s sex. “Household power and decision-making” is akin to “the sexual division of power” which refers to control, authority, and coercion within heterosexual relationships. Lastly, “other oriented” is similar to “the structure of cathexis,” which depicts the unequal roles and norms that dictate relationships between men and women.

We created the first domain, *gendered expectations*, as both men and women often talked about which household tasks men versus women actually do and should do, such as caring for children. A woman from a focus group said, “We have to take care of the children and bathe them and clean their clothes. That is the duty of the mother.” They also discussed which jobs outside of the home are appropriate for women versus men. A woman from a focus group said, “Hard labor which men can do; we women can’t do that. This is what prevails in the rural communities.” An item from the scale that reflects this sub domain is: “In most families you know, only men are the ones who earn money for the family.”

The second domain, *household power and decision-making*, refers to who is responsible for household finances and making decisions around which major household items to buy as these decisions were often gendered. It also refers to general autonomy for women, the need to obey one’s husband, and seeking permission to do things such as leave the house for any reason. A mother-in-law said, “Women generally don’t go alone outside. When they go, they go with their husbands.” A woman from a focus group said, “She must obey her husband and the other elders of the family.” An example of a scale item that we created within this sub domain is: “In most families you know, women ask permission from their husband or mother-in-law to leave the house.”

The third domain, *other oriented*, refers to the ways in which women put others before themselves. For example, women reported that they often eat “last” and whatever is “leftover” after the rest of the family has eaten. One woman from a focus group said, “after everyone has eaten, I pick up the utensils and clean their utensils and then I eat. I will give food to others first, but I will eat later.” Medical doctors and frontline health workers also reported that women “take care of their family before taking care of themselves.” A man from a focus group reiterated this message, “Always women are concerned about their children and husband, and they keep their own problems sometimes within themselves. They keep it suppressed.” An item from the scale that reflects these findings is: “In most families you know, women eat whatever is left over after the rest of their family has finished eating.”

Each of these larger domains was crossed with a descriptive norm component and a separate injunctive norm component to represent this dual aspect of social norms. This was particularly important because our qualitative findings illustrated both expectations around how a woman should act (injunctive norms; e.g., women must obey their husbands) and perceptions around how women do behave in the community (descriptive norms; e.g., women do not go alone outside). We chose individual questionnaire items based on prevalence and relevance in the transcripts. See Sedlander et al. (2021) for a full description of qualitative findings that informed item development.

**Literature Review and Expert Review.** In addition to primary data collection through the qualitative interviews, we also conducted a literature review on existing gender norms measures, including scales from different countries and continents, to improve external validity. To assess face validity, we gathered input on both the individual items and overall domains from five gender and social norms experts in the field and from our research partners in Odisha, India. We specifically chose researchers from different countries to ensure that the scale is representative of gender norms in India and can be used in other rural settings. Feedback from these experts (both local and international) centered on comprehension, cultural context, and item wording, and this process led to revisions to the scale. For example, we chose to make the scale a community-level scale (e.g., all items begin with the same reference group, “most families you know”) rather than mixing reference groups, such as also asking about husbands or mothers-in-law, which would have required many more questions. We also decided to mirror the descriptive and injunctive norms questions. In other words, for each descriptive norm item, we have the same item written as an injunctive norm. For example, in the descriptive norms subscale the item, “In most families you know, taking care of children is only the woman’s job” is also written in the injunctive scale with the following item, “Most families you know believe that it *should* only be the woman’s job to take care of the children.” This is a small but important difference to capture both descriptive and injunctive norms.

Originally, we wrote the questions in English and our research partners translated them to Odiya (the local language in Odisha, India). A second translator conducted quality assurance of the Odiya translation. Finally, a bilingual researcher discussed each question with the first author to ensure that the Odiya version represented the English version accurately.

**Cognitive Interviews.** After translation was complete, to refine the item pool and to assess content validity, we conducted eight cognitive interviews with women of reproductive age from neighboring villages. These interviews helped us to determine whether they interpreted the items as intended and whether they were being interpreted consistently among women with varying sociodemographic backgrounds (e.g., educational status, tribal versus non-tribal). During the cognitive interviews, we asked respondents each question individually and probed regarding interpretation and comprehension. We audio recorded each cognitive interview, transcribed them in Odiya, translated them to English, and held meetings to discuss findings. Based on findings from the cognitive interviews, we edited the questions, including removing words that did not translate well such as “leadership,” discussed the best word in Nepali to describe “community,” removed one item altogether, and added a new item. We also decided to add in questionnaire instructions for the interviewer to mark the end of the descriptive norms section and the beginning of the injunctive norms section in the following way:

Directions: Say, this section asks about what most of the people in your community (hamlet or village) believes people *should* do. This may not be what people are doing, but what others think they should be doing. For example, maybe in this community everyone knows that they should wash their hands with soap and water before they eat but they don’t do it every time. So, I will ask you questions about what the community expects, not what is actually done. Remember that there are no right or wrong answers, these are just your opinions, and nobody from this community (hamlet or village) will know how you responded.

#### Pilot Testing

We then pilot tested the gender norms questions with 36 women in nearby villages where we administered the final scale. We did this to avoid bias because we did not want the same women taking the pilot version as the final version. During the pilot testing phase, items were rated using a response scale ranging from 1 (*strongly disagree*) to 5 (*strongly agree*). Based on initial analysis from the pilot test, including examining the variance in question responses, we revised questions to improve clarity including making them all the same direction (all representing inequitable gender norms instead of switching back and forth between equitable and inequitable frames) and edited them to increase response variation. This process resulted in a final list of 28 items (see Table 1).

**Table 1:** Factor Loadings for the G-NORM Scale

	Original 28 Items		Reduced 18 Items	
	Pearson’s	Polychoric	Pearson’s	Polychoric
**Descriptive norms: “In most families that I know…”**				
1. Taking care of children is only the woman’s job.	0.43	0.59	0.44	0.59
2. Only men are the ones who earn money for the family.	0.50	0.53	0.50	0.54
3. Boys are more educated than girls.	0.40	0.40		
4. Women stop going to school after they get married.	0.06	0.08		
5. There are times when a husband beats (hits) his wife.	0.50	0.62	0.51	0.63
6. Women obey their husbands in all matters.	0.51	0.54	0.51	0.54
7. Only men make decisions about household income and expenses.	0.57	0.68	0.59	0.69
8. Women ask permission from their husbands to get medical treatment of any kind.	0.49	0.53	0.48	0.52
9. Husbands make the decision about buying major household items (e.g., television, refrigerator, bicycle, motor bikes).	0.48	0.64	0.48	0.64
10. Women ask permission from their husband or mother-in-law to leave the house.	0.27	0.29		
11. Women take care of their husbands, children, and in-laws before they take care of themselves.	0.28	0.39		
12. Women eat last, after all the family members have eaten.	0.44	0.51	0.43	0.50
13. Women eat whatever is left over after the rest of their family has finished eating.	0.66	0.77	0.66	0.76
14. Women do all of the housework and finish it before taking rest.	0.26	0.36		
**Injunctive norms: “Most families that I know believe that…”**				
15. It *should* only be a woman’s job to take care of the children.	0.51	0.59	0.52	0.60
16. Men *should* be the only ones who earn money for the family.	0.58	0.60	0.57	0.59
17. Boys *should* be more educated than girls.	0.47	0.47		
18. Women *should* stop going to school after they get married.	0.17	0.18		
19. Women *should* be beaten in certain circumstances.	0.62	0.70	0.64	0.72
20. Women *should* obey their husbands in all matters.	0.56	0.58	0.55	0.58
21. Only men should be responsible for household income & expenses.	0.63	0.75	0.64	0.76
22. Women should ask permission from their husbands to get medical treatment of any kind.	0.49	0.52	0.49	0.52
23. Husbands *should* make the decision about buying major household items (e.g., television, refrigerator, bicycle, motor bikes).	0.48	0.58	0.50	0.60
24. Women *should* ask permission from her husband or mother-in-law to leave the house.	0.28	0.30		
25. Women should take care of their husbands, children, and in-laws before they take care of themselves.	0.35	0.51		
26. Women *should* eat last, after all the family members have eaten.	0.49	0.56	0.48	0.55
27. Women *should* eat whatever is left over after the rest of their family has eaten.	0.67	0.74	0.68	0.74
28. A woman *should* do all of the housework and finish it before taking rest.	0.30	0.40		

#### Stage 2: Preliminary Psychometric Testing

We incorporated the entire pool of 28 scale items into the baseline data collection for the parent study. Women were eligible for inclusion in this study if they were between the ages of 15 and 49, spoke Odiya, lived in the data collection villages, and did not plan to move in the next year (as this was part of the baseline data collection for a longitudinal study). To recruit participants, we created a household listing of eligible women within the selected clusters. Once we determined the number of eligible women, we used a proportionate random sampling method to select households from the clusters to get our total sample. Research assistants read questions out loud to women and completed women’s responses via a tablet-based questionnaire in Odiya. They explained the purpose of the study, obtained written consent, and provided participants with a 100 rupee (the equivalent to about $1.50 U.S. dollars) stipend for their time. We collected data between July and September 2019. The overall sample size was 4,110 women.

To reduce respondent burden and thereby improve data quality, we used a planned missing data design for several components of the baseline questionnaire, including this scale. Planned missing data designs can increase the validity of the results as reduced time to complete the questionnaire can result in higher quality responses and therefore better-quality data (Little & Rhumtella, 2013). A planned missing design allowed us to collect incomplete data from participants by randomly assigning them to have missing items. Given that data were missing completely at random, missingness did not depend on either the observed or missing values. Therefore, this design conforms to the data missing completely at random assumption. Our design randomly divided the overall sample into four equally sized subsamples. The first subsample did not receive any of the 28 scale items. All the remaining 3,110 women received scale items 1, 5, 9, 13, 17, 21, and 25 – the anchor items for the planned missingness design. One third of the sample received items 2, 6, 10, 14, 18, 22, and 26; one third of the sample received items 3, 7, 11, 15, 19, 23, and 27; and one third of the sample received items 4, 8, 12, 16, 20, 24, and 28. Psychometricians recommend a minimum of 10 participants per item to produce reliable estimates (Nunnaly, 1978), and our sample far exceeded this minimum.

To account for our item-level missing data design, we used multiple imputation. Multiple imputation is a two-step approach in which each missing value is filled in with a set of imputed values based on responses to other questions, resulting in complete data sets (Graham et al., 2007; Schafer & Graham, 2002). In the imputation phase, we created multiple copies of the data, each with different imputed values. To impute the data, we included other auxiliary variables from the same questionnaire that provide additional information to impute the missing data. After performing multiple imputation, we pooled the estimates and standard errors into a single set of results. Based on Graham et al.’s (2007) rule of thumb to use 20 or more copies of the data, we ran 25 imputations to create our final imputed dataset.

**Fig. 1:**
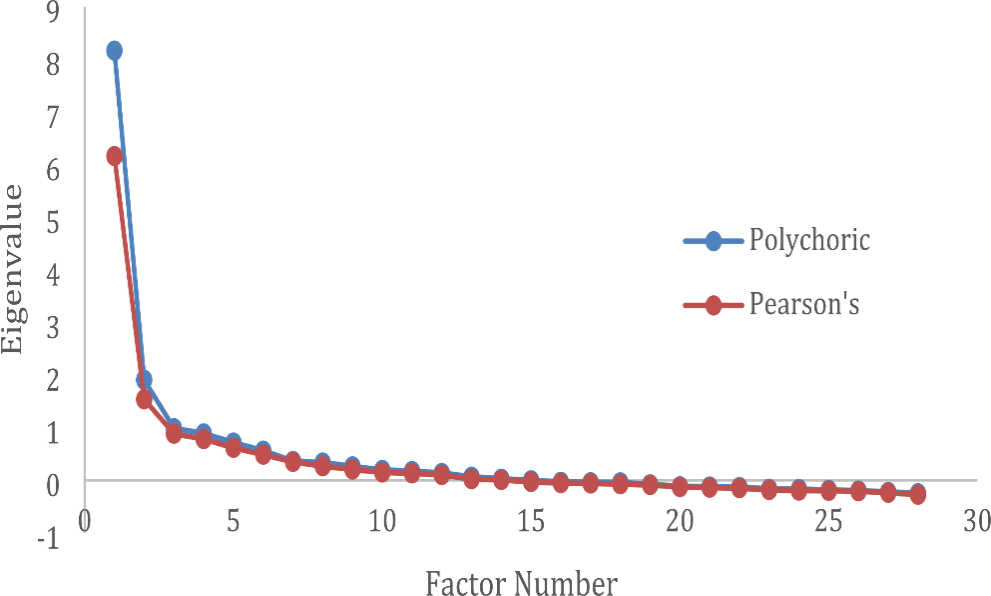
Scree Plot of Eigenvalues after Factor Analysis

To understand the scale’s psychometric properties and to check for multicollinearity, we examined the distributions and correlations for all items. To explore the variability in responses, we also examined the range, mean, and standard deviation of each item in our pool (DeVellis, 2017). We then ran exploratory factor analysis to determine the dimensionality of the scale and identify and remove poorly performing items. To do this, we obtained a single Pearson correlation matrix and a single polychoric correlation matrix using all 25 imputed datasets, and then used the principal factors method to extract the factors and determine their eigenvalues. We created scree plots of the eigenvalues and retained factors that had an eigenvalue of 2 or above. Factors that have an eigenvalue just over 1 are only slightly better than one item by itself (DeVellis, 2017, p. 166). We also visually inspected the scree plots to ensure that we extracted the correct number of factors (see Fig. 1). These analyses suggested a one-factor solution. We therefore reran the factor analysis constraining the number of factors to one and obtained the standardized factor loadings from this solution.

Next, following Comrey and Lee’s (1992) guidelines, we evaluated loadings on each factor and individually removed one item at a time that had a factor loading of less than 0.4 starting with the lowest factor loading, moving to the next lowest loading, and so on until we only kept items with a factor loading of 0.4 or higher (see Table 1). Based on social norms theory, we stipulated items must be removed in pairs (i.e., if the injunctive norm item involving a certain behavior is removed, then the corresponding descriptive norm item must also be removed, and vice versa). Table 1 shows the original pool of all 28 items with factor loadings.

#### Stage 3: Scale Validation

Our scale validation analyses used data from the RANI project’s end line survey, conducted between February and March 2021. All women who participated in the baseline survey were eligible to participate at end line, but 330 (8.0%) of the original 4,110 were lost to follow-up, leaving 3,780 available for analysis. Data collection procedures were very similar to those described above. No planned missingness design was used at end line, so all 18 of the remaining scale items were administered to all participants.

We conducted two sets of analyses using the resulting data. First, we conducted confirmatory factor analyses with two goals: to evaluate the fit of the one-factor model suggested by our analyses of the baseline data, and to compare the fit of that model to a two-factor model suggested by the theory of normative social behavior (Rimal & Lapinski, 2015). The latter was responsive to a call to develop gender norms measures with a “conceptual foundation consistent with social norms science” (Advancing Learning and Innovation on Gender Norms (ALIGN), 2021). Therefore, we compared a one factor model with a two-factor model (descriptive and injunctive norms as two separate subscales).

In these analyses, we first imposed the assumption of conditional independence, i.e., that all covariation between the items is attributable to the underlying factor or factors being measured. We then relaxed this assumption, first by allowing the errors / uniquenesses of analogous descriptive norms and injunctive norms items (e.g., items 1 and 15, items 2 and 16, and so on) to be correlated, and subsequently allowing for additional correlations between errors / uniquenesses as suggested by modification indices. Thus, we compare a total of six models: the one-factor model with no correlated errors, the two factor model with no correlated errors, the one factor model with error correlations between analogous pairs of items, the two factor model with error correlations between analogous pairs of items, the one factor model with those error correlations plus correlations between the errors on items 12 and 13 and items 26 and 27. We conducted these analysis in MPlus 8.4 because this software package incorporates a weighted least squares estimator that is optimal for indicators with ordinal response scales and produces key indices of model fit. To examine model fit we used the model chi-squared and degrees of freedom, the Bentler Comparative Fit Index (CFI), the Tucker-Lewis Index, Root Mean Square Error of Approximation (RMSEA), and Standard Root Mean Square Residual (SRMR). See Table 2 for acceptable ranges for each fit statistic.

**Table 2:** Factor Loadings and Model Fit Statistics from Six Confirmatory Factor Analysis Models (*n* = 3780)

Factor Structure	Single Factor Model			Two Factor (Descriptive and Injunctive) Model		
Correlated Errors	None	Analogous Pairs	Pairs plus 12 & 13, 26 & 27	None	Analogous Pairs	Pairs plus 12 & 13, 26 & 27
Fit Statistics						
RMSEA	0.270	0.268	0.236	0.241	0.206	0.183
CFI	0.785	0.803	0.849	0.830	0.884	0.911
TLI	0.757	0.760	0.813	0.806	0.858	0.889
SRMR	0.145	0.137	0.126	0.125	0.104	0.098
df	135	126	124	134	125	123
Chi-squared	9423.2	8656.7	6669.5	7465.4	5145.4	3990.3
Factor Loadings						
Item 1	0.555	0.528	0.548	0.621	0.620	0.631
Item 2	0.630	0.617	0.651	0.707	0.714	0.729
Item 5	0.604	0.583	0.600	0.664	0.657	0.665
Item 6	0.646	0.637	0.674	0.736	0.747	0.764
Item 7	0.693	0.667	0.691	0.775	0.766	0.779
Item 8	0.611	0.546	0.574	0.692	0.653	0.665
Item 9	0.579	0.564	0.577	0.639	0.640	0.645
Item 12	0.615	0.626	0.515	0.687	0.710	0.600
Item 13	0.667	0.669	0.575	0.745	0.753	0.654
Item 15	0.583	0.569	0.588	0.617	0.616	0.634
Item 16	0.722	0.719	0.744	0.752	0.755	0.775
Item 19	0.660	0.650	0.666	0.698	0.698	0.719
Item 20	0.772	0.775	0.805	0.812	0.818	0.846
Item 21	0.677	0.663	0.680	0.720	0.708	0.728
Item 22	0.668	0.619	0.641	0.699	0.660	0.678
Item 23	0.658	0.651	0.666	0.701	0.703	0.721
Item 26	0.753	0.766	0.561	0.792	0.812	0.620
Item 27	0.752	0.757	0.538	0.788	0.794	0.594
Correlation between the descriptive and injunctive norms factors				0.658	0.527	0.561

*Note.* Good-fitting models are indicated by a Tucker-Lewis (TLI) and Comparative Fit Index (CFI) equal to or greater than 0.90 and a Root Mean Square Error Approximation (RMSEA) less than 0.08, and standardized root mean squared residual (SRMR) less than 0.10. (Vandenberg and Lance, 2000)

**Fig. 2:**
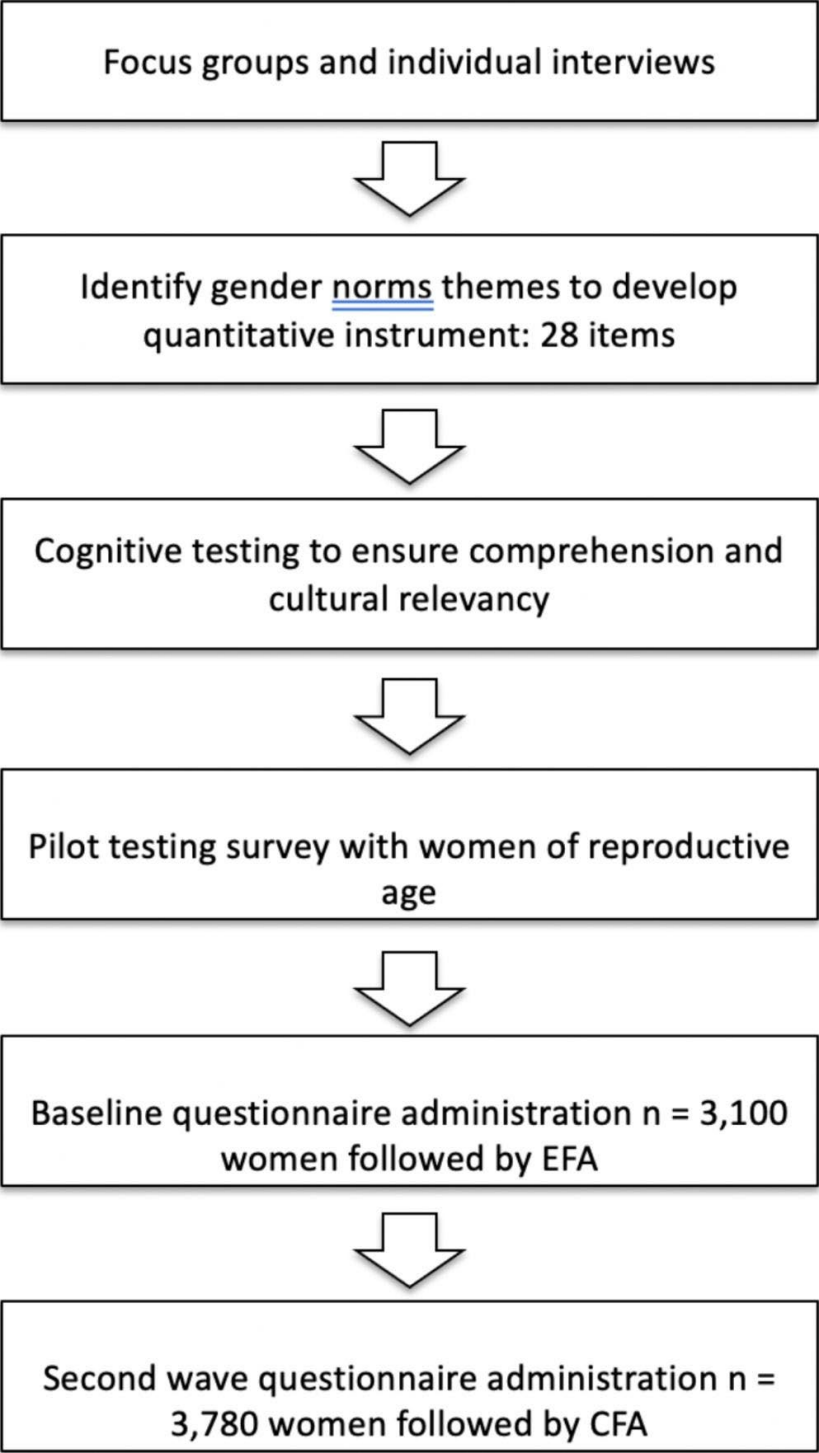
Stages to Develop and Validate the G-NORM

In addition to these confirmatory factor analyses, we sought to provide evidence for the validity of the scale using sub-group comparisons, in which one examines whether mean differences that are strongly expected a priori based on prior research and/or theory are in fact observed in the measure being examined. As previously stated, we hypothesized that gender norms would vary with age, education, tribal status, and residence in an intervention community (as opposed to a control community). To test these hypotheses, we generated an overall scale score as well as a perceived descriptive norms subscale score and a perceived injunctive norms subscale score. We then examined how mean values of these scores varied by age group, education group, membership in a Scheduled Tribe, and study arm. We reverse scored the gender norms questions to improve interpretability (higher scores = more equitable gender norms). We used linear regression models with dummy indicators of group membership to obtain overall *p*-values and *p*-values for all pairwise comparisons, with robust/clustered standard errors to account for the cluster sampling design (See Fig. 2 for a visual depiction of methods to develop and validate the G-NORM scale).

## Results

### Descriptive Statistics

Table 3 presents a description of the participants (*n* = 3110) whose baseline data contributed to the initial psychometric analysis. Approximately 1,000 participants were omitted because, according to the planned missingness design, they were not asked to answer any of the gender norms items. The sample included women with varying socio-demographic characteristics. The mean age was 30 years old (*SD* = 8.71). The modal level of education (55% of participants) was between Grades 6 and 12, with 18% of participants reporting no formal education and 3% who had attended any school beyond Grade 12. Almost all participants were Hindu and belonged to a caste, with more than half belonging to the “Other Backward Class” category, while 14% belonged to the Scheduled Caste, and 28% belonged to the Scheduled Tribe. Approximately 81% of women were married and 15% were single. Almost a quarter of the sample had no children, while just over half had one or two children. Only half of the sample owned a mobile phone.

**Table 3:** Description of the Baseline Sample (*N* = 3,110)

Variable	
Age (Mean, SD)	30.22 (8.71)
Education (%)	
No school	17.9
Completed up to class 5	23.7
Completed up to class 12	54.8
More than class 12	3.4
Religion (%)	
Hindu	99.8
Christian	0.1
Caste and Tribe Status (%)	
Scheduled Caste	13.6
Scheduled Tribe	28.1
Other Backward Class	56.0
None of them	2.1
Marital Status (%)	
Single	15.0
Married	80.7
Divorced	0.1
Separated	0.7
Widowed	3.2
Number of Children (%)	
None	23.5
One or two	55.6
Three or four	18.6
Five or more	2.1
Currently Pregnant (%)	
Yes	5.1
No	94.8
Own a Mobile Phone (%)	
Yes	49.0
No	50.9

### Initial Psychometric Analysis

Figure 1 presents scree plots from principal factor analyses of the Pearson and polychoric correlation matrices for all 28 gender norms items. Visual inspection of the plots along with application of the eigenvalue > 2 rule suggested a single factor solution. Factor loadings from the one-factor solution for all 28 items are presented in the first two columns of Table 1. Ten items had factor loadings of 0.40 or below according to analyses of both the Pearson’s and the polychoric correlation matrices. Removal of these ten items left 18 items, and principal factor analyses of the Pearson’s and polychoric correlation matrices of these remaining items appear in the third and fourth columns of Table 1. All 18 of these items had factor loadings above 0.40 in analyses of both matrices.

### Confirmatory Factor Analyses

Table 2 presents fit statistics from six confirmatory factor analysis models obtained via the weighted least squares estimator, as well as factor loadings, and for the two-factor models, the latent correlation between those factors. The first three columns show that the fit of the one-factor model improves with the addition of pairwise correlations among the item-specific errors. The addition of nine pairwise correlations among analogous descriptive and injunctive norms items decreases the RMSEA from 0.270 to 0.268, and the SRMR from 0.145 to 0.137; and increases the CFI from 0.785 to 0.803 and the TLI from 0.757 to 0.760. The addition of two more error correlations decreases the RMSEA and SRMR further to 0.236 and 0.126, respectively; and increases the CFI to 0.849 and the TLI to 0.813. Even for the third version of the model, however, these statistics are indicative of only a moderately well-fitting model. Across all six models, all factor loadings were adequate; the lowest loading for any item in any model was 0.515. In the final model, the descriptive and injunctive norms were moderately correlated, with a latent correlation of 0.561.

The fourth, fifth, and sixth columns of Table 2 show that the fit of the two-factor model, with separate factors for descriptive and injunctive norms, was substantially better. This was true whether we compare models with no correlations between item-specific errors, correlations only between analogous descriptive and injunctive norms items, or the full set of eleven pairwise correlations between item-specific errors. Thus, while our preliminary psychometric analysis of the baseline data led us to select a one-factor model, these analyses suggest that a two-factor model, with separate factors for items assessing descriptive and injunctive norms, provides a better fit. Even the two-factor model with all eleven pairwise correlations among errors, however, fits only moderately well (RMSEA = 0.183, CFI = 0.911, TLI = 0.889, SRMR = 0.098). The descriptive and injunctive norms scales had Cronbach’s alphas of 0.810 and 0.840, respectively, while the combined, 18-item scale had a Cronbach’s alpha of 0.870.

### Comparisons by Subgroups

Table 4 presents results of subgroups (i.e., age, education, caste, participation in the parent intervention) for the combined, descriptive, and injunctive norms scales. As hypothesized, younger age groups had higher mean scores on all three scales than older age groups. In other words, younger women who completed the scale reported more equitable gender norms among “most families they know” compared to their older counterparts. Omnibus F tests show that age-group differences were statistically significant for the overall scale (*p* = .0392) and the descriptive norms subscale (*p* = .0327). While the pattern of equitable gender norms decreasing with age was evident for the injunctive norms scale as well, it was not statistically significant (*p* = .0673). The next panel shows that, as hypothesized, higher education is associated with more equitable average gender norms for the overall (*p* < .0001), descriptive norms (*p* = .0001), and injunctive norms (*p* < .0001) scales. In contrast, membership in a Scheduled Tribe was not associated with average levels of the overall (*p* = .2813), descriptive norms (*p* = .2144), or injunctive norms (*p* = .4199) scales. Finally, and contrary to what we hypothesized, average scores were higher (and thus more equitable) in the control group (women who were *not* part of the larger parent intervention to increase iron folic acid supplement use) than in the treatment group (women who were not exposed to the intervention), but this difference was statistically significant only for the overall scale (*p* = .0270), and not for the descriptive norms (*p* = .2136) or injunctive norms (*p* = .0538) scales.

**Table 4:** Means and Confidence Intervals by Age, Education, Caste, and Parent Study Condition

	Total G-Norm Scale	Descriptive Norms	Injunctive Norms
Age Group Comparisons (*t*-tests)	Mean (95% CI)	Mean (95% CI)	Mean (95% CI)
1. 15–19 years	2.24 (2.05, 2.43)	2.02 (1.82, 2.22)	2.46 (2.22, 2.70)
2. 20–29 years	2.06 (1.97, 2.15)	1.79 (1.70, 1.88)	2.33 (2.18, 2.47)
3. 30–39 years	2.03 (1.94, 2.13)	1.79 (1.67, 1.90)	2.28 (2.13, 2.43)
4. 40 + years	1.96 (1.85, 2.08)	1.75 (1.67, 1.83)	2.17 (2.00, 2.35)
Comparison *p*-values			
Overall omnibus *F*-test	0.0392	0.0327	0.0673
2 vs. 1 (*t*-test)	0.0429	0.0086	0.2525
3 vs. 1 (*t*-test)	0.0235	0.0050	0.1403
4 vs. 1 (*t*-test)	0.0057	0.0045	0.0305
3 vs. 2 (*t*-test)	0.4363	0.9154	0.2283
4 vs. 2 (*t*-test)	0.0653	0.3427	0.0373
4 vs. 3 (*t*-test)	0.1310	0.3724	0.1616
Education Comparisons (*t*-tests)	Mean (95% CI)	Mean (95% CI)	Mean (95% CI)
1. None	1.92 (1.82, 2.02)	1.68 (1.57, 1.78)	2.16 (2.02, 2.31)
2. Class 1 to 5	1.89 (1.78, 2.00)	1.68 (1.56, 1.80)	2.09 (1.94, 2.25)
3. Class 6 to 12	2.14 (2.04, 2.24)	1.89 (1.79, 1.98)	2.39 (2.25, 2.54)
4. More than Class 12	2.49 (2.28, 2.71)	2.09 (1.87, 2.32)	2.89 (2.47, 3.31)
Comparisons *p*-values			
Overall omnibus *F*-test	0.0000	0.0001	0.0000
2 vs. 1 (*t*-test)	0.4468	0.9168	0.2260
3 vs. 1 (*t*-test)	0.0002	0.0001	0.0013
4 vs. 1 (*t*-test)	0.0000	0.0005	0.0011
3 vs. 2 (*t*-test)	0.0000	0.0002	0.0000
4 vs. 2 (*t*-test)	0.0000	0.0006	0.0004
4 vs. 3 (*t*-test)	0.0019	0.0576	0.0103
Tribal Status	Mean (95% CI)	Mean (95% CI)	Mean (95% CI)
Not Tribal (*t*-test)	2.06 (1.96, 2.15)	1.81 (1.71, 1.92)	2.30 (2.15, 2.45)
Tribal (*t*-test)	2.00 (1.89, 2.10)	1.75 (1.66, 1.85)	2.24 (2.09, 2.40)
Comparison *p*-values	0.2813	0.2144	0.4119
Study Arm	Mean (95% CI)	Mean (95% CI)	Mean (95% CI)
Control (*t*-test)	2.13 (2.02, 2.24)	1.85 (1.73, 1.97)	2.41 (2.23, 2.58)
Treatment (*t*-test)	1.95 (1.84, 2.06)	1.74 (1.61, 1.87)	2.16 (1.97, 2.34)
Comparison *p*-values	0.0270	0.2136	0.0538

***Note.*** Higher means show more equitable gender norms. Confidence Interval = CI

## Discussion

This study used a mixed-methods approach to develop and validate a theory-based gender norms scale, the G-NORM scale. Our findings indicated that two subscales, with one factor each, comprising nine items each, best represented the overall construct of descriptive and injunctive gender norms. As hypothesized, we also found that younger, more educated women reported greater endorsement of equitable gender norms than their counterparts.

The G-NORM scale contributes to a growing interest in adequately measuring gender norms (EMERGE, 2021; ALIGN, 2021). Our study expands on past work in at least three ways. First, this scale includes both descriptive and injunctive norms as two separate subscales to allow researchers to identify and disentangle their potentially distinct roles in attitudes and behaviors. Second, this scale measures perceptions of community-level norms instead of individual attitudes or beliefs by asking about the attitudes and beliefs of others in their community. Finally, the scale items represented broader domains of gender norms, including norms around being other oriented, gendered expectations, and household power and control. While the final scale does not include three separate sub domains as theorized in Connell’s theory of gender and power, the single factor structure within the descriptive and injunctive norms subscales are consistent with the overlapping nature of the three domains. Items from each of the original three sub domains are included in the final scale (see Table 1 for a comparison of original and final scale items).

Lastly, this is a parsimonious scale as researchers can decide to use one subscale or both subscales (descriptive gender norms and injunctive gender norms) to measure the complex construct of gender norms. Given resource constrained projects and on the ground realities, a parsimonious scale with two subscales may make the difference between whether researchers choose to measure gender norms as part of their study at all. In our sample, the injunctive norms subscale had more variance and younger women reported that injunctive norms (perceived social expectations) were more equitable than descriptive norms (perceived actual behavior). While the two subscales, descriptive and injunctive norms, are significantly correlated (*r* = .56 *p* < .001), they are not so correlated as to suggest multicollinearity (Kim, 2019). A correlation closer to or above 0.90 could be multicollinearity, but they are clearly covering two distinct and important concepts and separating them out will allow researchers to examine which subscale is a better predictor of behavior change.

We hypothesized that the parent intervention, where we administered the G-NORM scale, would increase gender equitable norms in treatment communities compared to control communities given that we collected data at baseline and end line. However, we found the opposite for the full G-NORMS scale: women in the treatment communities reported less equitable gender norms on average than did women in control communities. One plausible explanation for this is that the intervention served to heighten awareness of inequitable gender norms among women in treatment communities. This is an inherent complication of measuring norms. An intervention focused on changing gender norms may serve to raise awareness about inequitable gender norms thereby increasing perceptions that gender norms are less equitable within a community. Therefore, it can be difficult to measure the impact of the intervention. If possible, a more objective measure such as number of women in the workforce, number of women attending school, etc. may be necessary to measure in addition to perceptions of gender norms.

### Limitations and Future Research Directions

Our study has some limitations that may impinge upon the interpretation of the results. Specifically, unlike other social norms scales, this scale is focused on individual perceptions of community beliefs and behaviors (rather than individual beliefs and behaviors). While including individual perceptions about community level norms is a strength of the methods, we did not compare perceived community level norms to individual attitudes and beliefs to empirically show that we are tapping into two different concepts. We relied on theory. Moreover, participants may feel uncomfortable or not knowledgeable enough to respond based on the community norms rather than their own individual attitudes and behaviors. Furthermore, while we asked about village level norms, we only included one referent group (the community). Asking about other referent groups like husbands or mothers-in-law may have produced different results.

Additionally, these data, like all self-reported data, are vulnerable to social desirability bias. To reduce these biases, we trained data collectors and included a script stating that there are no right or wrong answers and we were asking for their opinion on attitudes and behaviors in the community to the best of their ability. Furthermore, we only tested this scale in two blocks in one rural district of India among women of reproductive age. Testing this scale in another context and including men would add to the validity of the scale. Finally, the fit of the final two-factor model in our confirmatory factor analyses was only moderately good, not excellent, leaving some concern about the underlying theoretical model. Future research that attempts to replicate this scale in other settings may want to investigate this further.

Despite these limitations, our scale has several strengths that set it apart from previous gender norms scales. Our scale is grounded in theory, which added clarity when we selected items for the scale. We also conducted extensive qualitative research among women of reproductive age and their reference groups (husbands, mothers-in-law, and key informants) in the community. We used findings from the qualitative research to inform the scale domains and items. Another strength of this study design is the large sample size, 3110 women at wave one and 3780 women at wave two. A scale from a large sample size increases the generalizability of our factor analysis findings (DeVellis, 2017, p. 203–204). We also conducted, audio recorded, and translated cognitive interviews and then subsequently pilot tested the questionnaire. Furthermore, operating from the level of individual perceptions of community gender norms (rather than individual perceptions) provides valuable information to the social norms field. Additionally, asking what others believe not what the individual participants believe may reduce social desirability bias. Lastly, we used a rigorous random sampling design to recruit all participants improving representativeness of the community.

These strengths improve both the internal and external validity of this scale. While context matters a great deal as gender norms vary widely, this scale may be relevant in other rural areas of India and in other South Asian rural contexts such as Nepal, Bangladesh, Pakistan, Sri Lanka, and Bhutan. It could also be applicable after some cognitive testing in other rural areas of low- and middle-income countries. Future research could psychometrically test and adapt this scale to new countries and continents.

### Practice Implications

Beyond the utility of providing an improved scale to the field, this paper shows that measuring gender norms before a behavioral intervention can help researchers understand the social context in which they are working. Past research shows that gender norms affect a myriad of behaviors including contraceptive use, iron folic acid supplement use, nutritional food intake, HIV testing, and immunizations (Caal et al., 2013; Cianelli, Ferrer, & McElmurry; Sedlander et al., 2021; Garrett & Barrington, 2013; Paudel et al., 2018; Singh et al., 2013). Given this, researchers should consider measuring gender norms before implementing a behavioral intervention to examine how they may be a barrier to behavior change.

Additionally, our comparison of responses to the gender norms questions (our tested hypotheses) can help interventionists understand how gender norms differ among sub populations so that they can tailor interventions appropriately. Specifically, our finding that women across age and education levels reported more equitable injunctive norms compared to descriptive norms illustrates that perceived expectations are changing faster than perceived behaviors around gender norms. This has implications for interventions trying to change gender norms as it is important to measure both expectations and real behaviors. Our scale with separate sub domains allows researchers to do just that.

## Conclusion

This scale improves on previous gender norms scales in three important ways. We include both descriptive and injunctive norms, we move beyond aggregating individual attitudes to measuring perceptions of community-level norms, and we expand on past sub domains included in gender norms measurement. Given that many gender researchers have stated that gender norms are not an individual construct but part of a larger social structure that includes perceptions about what others are doing and expectations for what one should do, it is critical to capture this higher-level factor. This scale not only provides perceptions of community level gender norms but also allows researchers to examine if descriptive versus injunctive norms are changing at different rates. Finally, it is essential to improve measurement to truly understand if and how gender norms are changing and if interventions are successfully changing gender norms, a known pathway to many important health behaviors.

## Data Availability

Data will be available in the George Washington University Data Repository – GW ScholarSpace: https://scholarspace.library.gwu.edu/?locale=en

## Code Availability

Code for this analysis can also be found in the George Washington University Data Repository – GW ScholarSpace: https://scholarspace.library.gwu.edu/?locale=en
